# Helmholtz’s Treatment in Hay-Fever

**Published:** 1874-10

**Authors:** 


Helmholtz's Treatment in Hay Fever.—Professor Binz, of Bonn, writes to "Nature" that from what he has observed of recent English publications on the subject of hay fever, he is led to suppose that the English authorities are not accurately acquainted with the discovery of Professor Helmholtz in 1868, that certain uncommon low organisms are present in the nasal secretions in this complaint, and that quinine arrests their action. Professor Helmholtz had been subject to this disease since 1847, and from the fact that the the attack always commences in his case between May 20 and the end of June, he was led to the suspicion that organisms might be the cause of the mischief. Examination with the immersion lens of a very good microscope, proved the presence of vibrio-like bodies, which he could never find at other seasons in the nasal secretions. Remembering the poisonous action of quinine upon infusoria, he made a weak neutral solution of sulphate of quinine, containing one part of the salt to eight hundred of water. This was effective enough, and caused moderate irritation of the mucous membrane. Lying flat on his back, he poured from a pipette about four cubic centimeters into the nostrils, turning the head about in order to let the liquid flow in all directions. This produced the desired effect. He could expose himself to the sun without bringing on the fits of sneezing and the other disagreeable symptoms; and it was sufficient to repeat the treatment three times a day even under the most unfavorable circumstances. One treatment a day is enough if he goes out only in the evening. No vibrios are then found in the secretion. After treatment for some days the symptoms disappear completely, but the treatment must be kept up until the end of the time during which the attack would ordinarily make its appearance. In Professor Helmholtz's case this is, as before said, between May 20 and June 30.—*Galaxy.*

